# The British Hospitals Association Conference

**Published:** 1910-09-17

**Authors:** 


### THE BRITISH HOSPITALS ASSOCIATION CONFERENCE.

### SOME IMPORTANT PROBLEMS OF THE DAY.

The British Hospitals Association is to be congratulated on the excellent programme that has been drawn up for its first annual meeting, which will be held at Glasgow on September 29 and 30 next. It will be remembered that this Association is the rejuvenated and in a sense reincarnated Hospitals Association which, founded in 1884, was responsible for much good work while it was an active organisation. Last year a vigorous effort was made to re-establish the Association on a new basis. It was felt that at the present moment, more than at any other, the necessity for organised institutional opinion, authoritatively announced through a body of experts who are in daily touch with hospitals in the British Empire, is urgent. There are many problems that await solution in which the voluntary hospitals are gravely interested, and it is of the utmost importance that the views of those who are best qualified to speak on behalf of these institutions should possess the added weight and influence which membership of a representative professional organisation carries with it.

### What the Association Means.

The attempt to restart the old organisation, under the new and, we venture to think, more appropriate, title of "The British Hospitals Association," has been followed with most conspicuous success. It is gratifying to be able to record that the membership of the Association is daily increasing, and that the new society may now be looked upon as being thoroughly representative of institutional interests in this country. The objects of the Association are to facilitate the consideration and discussion of matters connected with hospital management, and, where advisable, to take measures to further decisions arrived at, and to afford opportunities for the acquisition of a knowledge of hospital administration, both lay and medical. The President of the Association is Mr. Cosmo Bonsor, the President of Guy's Hospital, and until lately its Treasurer, whose reputation as a hospital worker is known to all our readers. The Honorary Treasurer is Mr. Conrad Thies, of the Royal Free Hospital, while Mr. William West and Dr. D. Mackintosh, of the Glasgow Western Infirmary, have undertaken the duties of Secretaries to the Association. The Council of the newly formed body is both influential and representative, including such well-known names in the hospital world as Sir Henry Burdett, Mr. H. J. Collins, Mr. Hayes, Mr. J. Hedderwick, Mr. Stewart Johnson, Mr. Charles Lupton, Mr. Glenton Kerr, Dr. Munro Moir, Sir Cooper Perry, Mr. Henry Peterkin, Mr. Roxburgh, and Mr. W. A. Tait.

### The Conference Programme.

We have received an advanced proof of the programme for the Conference. The meetings will be held in the University Buildings at Glasgow, and members may look forward to an interesting list of attractions, both intellectual and social. On the first day—Thursday, September 29—the Conference opens at ten o'clock in the forenoon, when the Hon. the Lord Provost (Mr. A. M'Innes Shaw) will welcome the delegates. The first paper will be read by Mr. Loch, the Secretary of the London Charity Organisation Society, and will deal with "The Majority Point of View on the Poor Law as regards General and Special Hospitals"—a subject which is likely to lead to an instructive and interesting discussion. This will be followed by a paper on "The Abuse of the Hospital and its Cure," by Mr. A. Scott Finnie, Treasurer of the Aberdeen Royal Infirmary, another subject that ought to give rise to an animated debate. On the following day Mrs. Sydney Webb will address the Conference, taking as her subject "A Unified County Medical Service and how it will affect the Voluntary Hospital," the address to be followed by a discussion. The further items on the day's programme include "The Institutional Treatment of Tuberculosis," by Dr. Nathan Raw, of the Liverpool Mill Road Infirmary, and a series of highly interesting questions mooted for general discussion. Among these latter are "What are the best Ambulance Arrangements for Hospitals? Is the Motor Ambulance quite Reliable?" "Should General Hospitals admit Private Patients? and, if so, what arrangements should be made for them?" "Should Private Rooms be provided in Fever Hospitals for Paying Patients? and can these latter be attended by their own medical men?"

### The Problem of Medical Relief.

It will be seen from this short summary that there is plenty of material provided for a thoroughly instructive and elucidative series of discussions on subjects that may be considered as being of primary importance to institutional workers at the present time. The outstanding interest of the Majority and Minority Reports of the Poor Law Commissioners has been adequately recognised by providing discussion on papers based on two widely divergent views of the question. The whole question of medical relief and reform will come up for debate, and it is hoped that it will be thoroughly threshed out. Everyone who is interested in the matter should make a point of attending the Conference and following the discussion. Again, the ever-present problem of hospital abuse is one of the most generally interesting that the Conference can tackle. At the same time it must not be forgotten that among the most interesting questions before the Conference will be some of those catalogued under the title of "Question Box"; for instance, the matter of paying patients in general and fever hospitals and their relations to the institutional staff. All these points demand a full and searching ventilation, and we look forward to a rich harvest of opinion and practical suggestion from the discussions. The social programme is as attractive as the meetings are likely to be. There will be various receptions and visits to the leading institutions in the City which are likely to prove of interest to members attending the Conference. Glasgow, we may point out, has the reputation of being one of the most "architectural" of Scottish cities, and from a construction and purely institutional point of view members will find plenty of interesting material for study, not only in such fine blocks as the Royal and Western Infirmaries and the Cancer Hospital, but in the various municipal and in many commercial buildings as well. The Lord Provost and Corporation of the City will entertain members attending the Conference to a luncheon to be held in the City Hall, George Square, on Friday, September 30.

The Hon. Secretaries will be glad to receive applications for membership before the Conference meets in order that the necessary arrangements may be made. Trustees, members of committees, or executive or professional heads of hospitals are eligible for membership. The annual subscription is half a guinea. Members desiring to bring forward any subjects for special discussion are requested to communicate with the Secretaries.

The official programme of the Conference has been attractively got up, and is well illustrated with photographs and views showing the principal buildings of institutional interest in Glasgow.

